# Linguistic adaptation and psychometric evaluation of Italian version of children’s sleep habits questionnaire

**DOI:** 10.1186/s13052-021-01119-z

**Published:** 2021-08-09

**Authors:** Melissa Borrelli, Iris Scala, Paola Festa, Dario Bruzzese, Ambrosina Michelotti, Elena Cantone, Adele Corcione, Martina Fragnito, Vincenzo Miranda, Francesca Santamaria

**Affiliations:** 1 Department of Translational Medical Sciences, Pediatric Pulmonology, Federico II, Naples, Italy; 2 Unit of Odontology, Bambino Gesù Children’s Hospital, IRCCS, Rome, Italy; 3 Department of Public Health, Federico II University, Naples, Italy; 4 School of Orthodontics, Department of Neurosciences, Federico II University, Naples, Italy; 5 Department of Neuroscience, Reproductive and Odontostomatologic Sciences, Ear Nose Throat Section, Federico II University of Naples, Naples, Italy

**Keywords:** Children’s sleep habits questionnaire, CSHQ, Sleep behavior, Children, Reliability, Italy

## Abstract

**Background:**

The Children’s Sleep Habits Questionnaire (CSHQ) is a parent-report questionnaire used to examine sleep behavior in children. Linguistic adaptation of CSHQ into several languages and/or psychometric analysis of reliability have been published.

**Main text:**

Our aim was to translate the original 33-items CSHQ from English to Italian and to examine its reliability for use in 4–10 years-old children of Italy. After translation and back-translation procedure of the original CSHQ, the Italian CSHQ (CSHQ-IT) was administered to 69 mothers of healthy children. Reliability of CSHQ-IT was examined by the internal consistency of the scale (using the Cronbach’s alpha coefficient), and by the test-retest analysis obtained by asking mothers who had completed the CSHQ-IT at baseline to re-complete it after a two-week interval (measured with the Lin’s Concordance Correlation Coefficient, CCC). The CSHQ-IT showed adequate internal consistency (Cronbach’s alpha = 0.81 for the total scale). The total CSHQ-IT score showed a strong correlation in retests (CCC 0.87; 95% Confidence Interval, 0.80; 0.92).

**Conclusion:**

CSHQ-IT is a valuable tool for evaluating sleep behavior in children 4–10 years-old in Italy. Future research should be implemented to provide definitive validity of CSHQ-IT in children with sleep-disordered breathing.

## Introduction

Sleep-disordered breathing (SDB) represents a continuum of high prevalent disorders, ranging from primary snoring to obstructive sleep apnea syndrome (OSAS) [1]. OSAS significantly affects quality of life of children and families, hence the scientific interest on the parent-report sleep questionnaires to screen pediatric OSAS has increasingly risen [2]. Currently, several questionnaires such as the Obstructive Sleep Apnea-18 (OSA-18 [3];), the Children’s Sleep Habits Questionnaire (CSHQ [4];), the Sleep Disturbance Scale for Children [5] and the Pediatric Sleep Questionnaire (PSQ [6];) are in circulation. Translation from the original English into other languages are available, CSHQ, PSQ, and OSA-18 being the most frequently translated tools. However, neither linguistic adaptation nor psychometric data are always available or described in detail [2].

The CSHQ, a retrospective, 33-items parent questionnaire, was developed to examine sleep behavior in 4–10 years-old US children [4]. The CSHQ questions generate a total Sleep Disturbance Scale which includes 8 subscales: (1) Bedtime Resistance, (2) Sleep Onset Delay, (3) Sleep Duration, (4) Sleep Anxiety, (5) Night Wakings, (6) Parasomnias, (7) Sleep Disordered Breathing, and (8) Daytime Sleepiness. The CSHQ has been widely used in many countries, and linguistic adaptation and/or validation procedure have been published [7–11]. In a study of cosleeping using CSHQ, Cortesi and coworkers assessed parents’ report on Italian healthy school-aged children’s sleep and behavioral problems [12]. The study showed adequate internal consistency of the CSHQ (Cronbach’s alpha = 0.66), but translation into Italian and test-retest details were not available. The aim of the current study was to translate the original 33-items CSHQ from English to Italian and to examine its reliability for use in the pediatric population of Italy.

## Methods

**Fig. 1:**
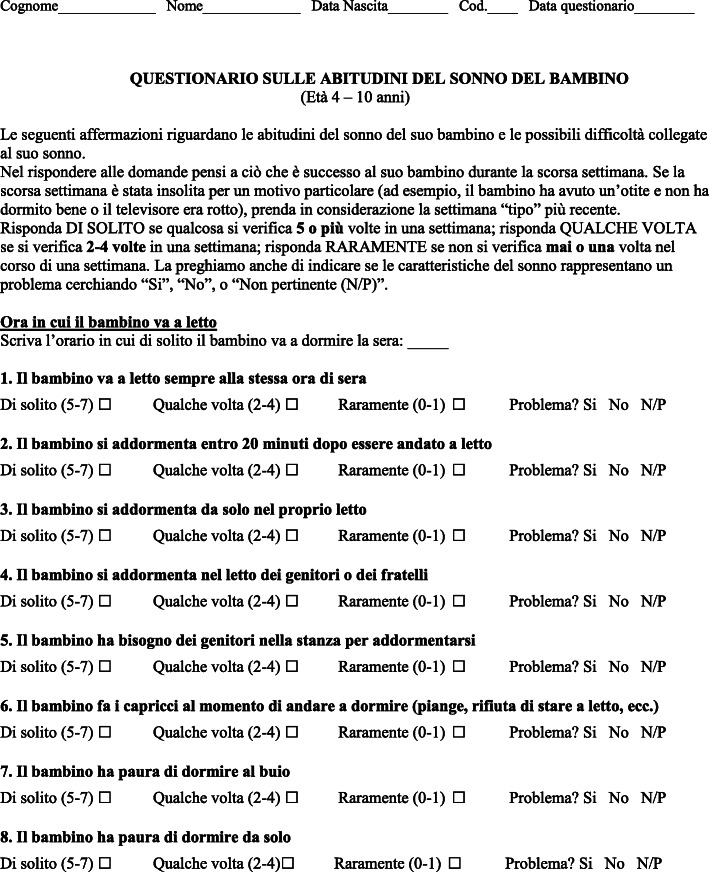
Italian version of the Children’s Sleep Habits Questionnaire (33-item version; CSHQ-IT)

The English original version of the 33-items CSHQ original questionnaire [4] was first translated into Italian, and the translation was reversed into English, and then again into Italian by an independent bilingual Italian-English speaker who was Italian mother-tongue, a pediatric pulmonologist (F.S.) and a pediatric orthodontist (P.F.) The 3 versions were unified by consensus between the translator and the physicians. The final version was back-translated by an independent bilingual Italian-English speaker who was English mother-tongue. After back-translation, a committee including pediatric pulmonologists (M.B., A.C.), a pediatric Ear, Nose, Throat specialist (E.C.), a pediatric orthodontist (A.M.) and a pediatric geneticist (I.S.) *plus* one expert translator, Italian mother tongue, adapted the Italian questionnaire to solve the small discrepancies and provided an Italian version that was equivalent to the original version and understandable to parents with low literacy level. In order to assess possible difficulties in understanding the questionnaire, the CSHQ Italian version was first tested by the same investigator (M.F.) on a sample of 15 volunteers recruited among the mothers of children scheduled for urological procedures at our department. Children neither had genetic/mental/psychiatric/systemic disease nor were receiving medication with effects on sleep. After some aspects were modified to facilitate comprehension, the translation into Italian was finalized. The Italian CSHQ questionnaire (CSHQ-IT; Fig. 1) was then administered to a total population of 69 mothers of healthy children (4–10 years-old) followed-up at a primary care pediatrician office. In the current study, the CSHQ-IT was administered only to the mother, who usually spends more time with the children than the father. Criteria for inclusion in the study included the informed consent signed by mother, the ability to read and understand Italian, and the full completion of the questionnaire. Children were excluded in case of age not included in the 4–10 years range, if any genetic, mental, psychiatric, or systemic disease were reported, if children were taking any medication with effects on sleep, or if mothers were unable to read and understand Italian or denied participating in the study. As in the original CSHQ scale, the frequency of sleep-related behaviors in the last previous week was rated on a 3-point scale as ‘usually’ (5 to 7 times per week, scored as 3 points), ‘sometimes’ (2 to 4 times per week, scored as 2 points) or ‘rarely’ (0 to 1 time per week, scored as 1 point). The total score was the sum of the responses obtained on each item and the highest scores indicated the worst sleep habits. CSHQ-IT was administered from September 2019 to February 2020.

### Statistical analysis

Reliability of CSHQ-IT was examined by two approaches: (a) the internal consistency of the scale, using Cronbach’s alpha coefficient for the total scale and for each subscale; and (b) the test-retest analysis, which was obtained by asking mothers who had completed the CSHQ-IT questionnaire at baseline to re-complete it after a two-week interval (i.e. a time gap long enough to ensure that participants would not remember their responses, but short enough to avoid significant changes in the sleep behavior). Test-retest reliability was measured using the Lin’s Concordance Correlation Coefficient (CCC) and examined the relationship between baseline and two-weeks later scoring. All statistical analyses were conducted using the statistical Platform R (vers. 4.0.1).

The study was approved by the Ethical Committee, Federico II University, Naples (protocol no. 104/19), and parents signed an informed consent.

## Results

**Fig. 2:**
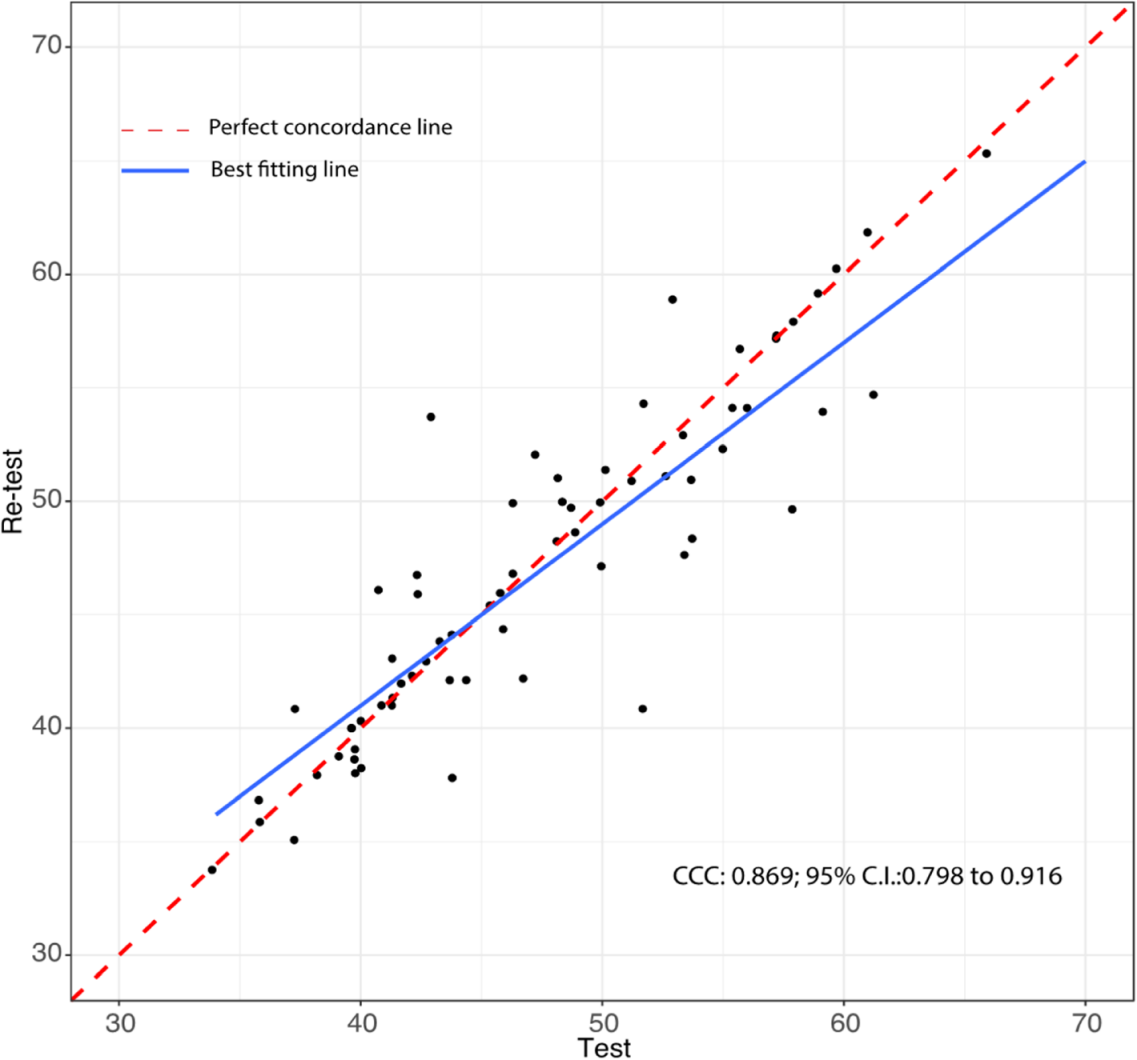
Test retest reliability measured by the Lin’s Concordance Correlation Coefficient (CCC; 95% Confidence Interval, CI) to examine the relationship between baseline and two-weeks later scoring of the total scale of CSHQ-IT

Completion of the CSHQ-IT requires 4 to 6 min’ time. All the enrolled mothers correctly completed the CSHQ-IT at baseline and after 2 weeks. The Table 1 summarizes the results of the internal consistency of the scale and of the test-retest analysis. The Cronbach’s alpha coefficient for the total scale was 0.81, and for the different subscales ranged from 0.45 (Night wakings) to 0.71 (Bedtime resistance)**.** Apart from Night wakings (0.45) and Parasomnias (0.48), most αs were moderate (Bedtime resistance 0.71, Sleep duration 0.70, Sleep anxiety 0.55, Sleep disordered breathing 0.69, Daytime sleepiness 0.59). The CCC for the total scale was 0.87 (95% CI, 0.80; 0.92; Fig. 2), and for the different subscales ranged from 0.51 (Sleep onset delay) to 0.88 (either Bedtime resistance or Sleep anxiety). Apart from the low CCC value for Sleep Onset Delay (0.51), all other subscales CCC values were > 0.6.

**Table 1:** Subscale Cronbach’s α and Concordance Correlation Coefficient (CCC) in the study population

SUBSCALE	Cronbach α	CCC (95% CI)
**Bedtime resistance**	0.712 (0.623;0.801)	0.886 (0.823; 0.928)
**Sleep onset delay**	NA	0.513 (0.319; 0.665)
**Sleep duration**	0.705 (0.591; 0.819)	0.752 (0.628; 0.839)
**Sleep anxiety**	0.55 (0.4; 0.7)	0.886 (0.823; 0.928)
**Night wakings**	0.455 (0.257; 0.653)	0.751 (0.629; 0.837)
**Parasomnias**	0.487 (0.333; 0.641)	0.846 (0.766; 0.9)
**Sleep-disordered breathing**	0.697 (0.595; 0.798)	0.859 (0.783; 0.91)
**Daytime sleepiness**	0.591 (0.46; 0.722)	0.854 (0.774; 0.907)

Abbreviations: *95% CI* 95% Confidence Interval, *NA* not applicable, subscale consists of 1 item

## Discussion

Despite the American Academy of Pediatrics recommends overnight PSG as the gold standard for the diagnosis of OSAS in children with SDB [1], access to PSG is still difficult or precarious in several countries. Hence, the evaluation of pediatric OSAS through parent questionnaires is of great clinical importance, with a low operational cost. Of all the published parent-report sleep questionnaires, the CSHQ has been applied more than any other in different countries as a screening tool of SDB [2]. To our knowledge, this study first provides reliability data of the Italian translation of the CSHQ. Our findings show that CSHQ-IT is a valuable instrument for evaluating sleep behavior in children 4–10 years-old. According to the results, Cronbach’s alpha coefficient for the total scale was 0.81. Most previous studies also including different sample sizes have shown similar internal consistency data, yet the number of study population has no influence on Cronbach’s alpha coefficients. Our results regarding internal consistency of the CSHQ-IT are in accordance with those from several previous studies made either in the US (Cronbach α ranging from 0.36–0.70) [4] or in Europe (i.e., the Dutch study: Cronbach α ranging from 0.47–0.68; the German study: Cronbach α ranging from 0.23–0.70; the Portuguese study: Cronbach α ranging from 0.44–0.74 [8–10]. Thus, the reliability of the CSHQ-IT could be considered adequate. The total CSHQ-IT score showed a strong correlation in retests.

The study has strengths and limitations. First, the novel information on reliability of CSHQ in Italian was provided, that was lacking in the literature. A limitation of our study is that we did not assess CSHQ-IT validity by comparing it to diagnostic procedures such as polysomnography (PSG). Finally, sensitivity and specificity of CSHQ-IT for detecting OSAS could not be evaluated.

In conclusion, our study shows that the CSHQ-IT has satisfactory internal consistency and reliability, is equivalent to the original English version and can be considered a valuable sleep-screening measure for both clinical and research uses. Future research hopefully focused on the relationship between CSHQ-IT and main indicators from PSG and/or other instrumental procedures should be implemented to provide definitive validity of CSHQ-IT in a pediatric population with a high probability of OSAS.

## Data Availability

Not applicable.

## Abbreviations

SDB: Sleep Disordered Breathing
OSAS: Obstructive Sleep Apnea Syndrome
OSA-18: Obstructive Sleep Apnea-18
CSHQ: Children’s Sleep Habits Questionnaire
PSQ: Pediatric Sleep Questionnaire
CSHQ-IT: Italian CSHQ questionnaire
CCC: Concordance Correlation Coefficient
PSG: Polysomnography
