# Correction to: Linguistic adaptation and psychometric evaluation of Italian version of children’s sleep habits questionnaire

**DOI:** 10.1186/s13052-021-01159-5

**Published:** 2021-11-03

**Authors:** Melissa Borrelli, Iris Scala, Paola Festa, Dario Bruzzese, Ambrosina Michelotti, Elena Cantone, Adele Corcione, Martina Fragnito, Vincenzo Miranda, Francesca Santamaria

**Affiliations:** 1 Department of Translational Medical Sciences, Pediatric Pulmonology, Federico II, Naples, Italy; 2 Unit of Odontology, Bambino Gesù Children’s Hospital, IRCCS, Rome, Italy; 3 Department of Public Health, Federico II University, Naples, Italy; 4 Department of Neurosciences, School of Orthodontics, Federico II University, Naples, Italy; 5 Department of Neuroscience, Reproductive and Odontostomatologic Sciences, Ear Nose Throat Section, Federico II University of Naples, Naples, Italy

**Correction to: Italian Journal of Pediatrics 47, 170 (2021)**

**https://doi.org/10.1186/s13052-021-01119-z**

The original article [1] mistakenly cropped Fig. 1.

**Fig. 1:**
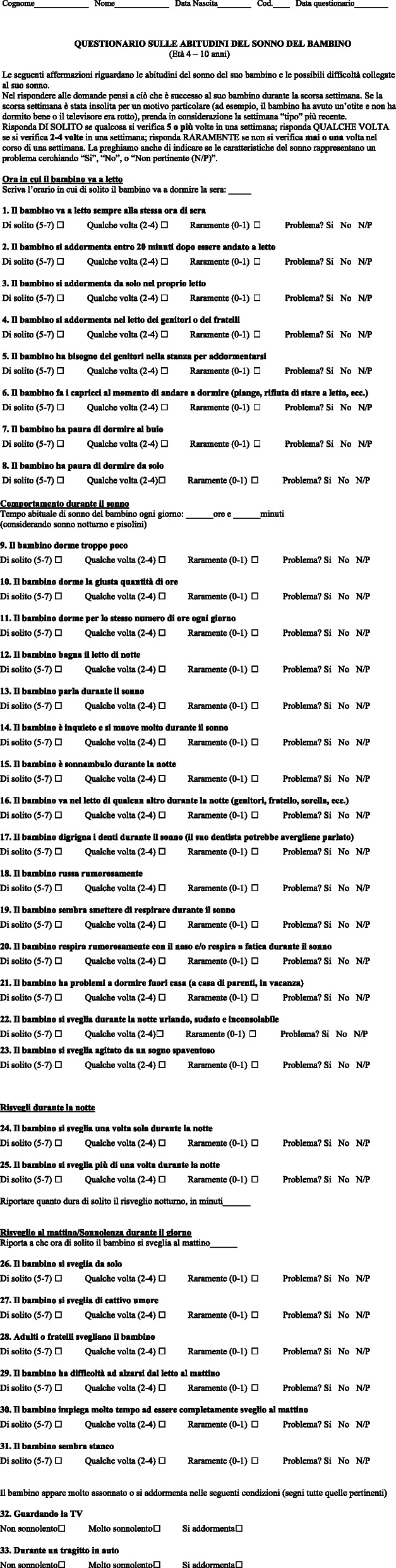
Italian version of the Children’s Sleep Habits Questionnaire (33-item version; CSHQ-IT)

The full, corrected version of Fig. 1 can instead be viewed ahead.
